# A newly identified lncRNA H1FX-AS1 targets DACT1 to inhibit cervical cancer via sponging miR-324-3p

**DOI:** 10.1186/s12935-020-01385-7

**Published:** 2020-07-31

**Authors:** Xinghua Shi, Jinzhi Huo, Xingping Gao, Hua Cai, Weipei Zhu

**Affiliations:** 1 Department of Obstetrics and Gynecology, the Second Affiliated Hospital of Soochow University, No. 1055 Sanxiang Road, Suzhou, 215000 China; 2 Department of Obstetrics and Gynecology, Qidong People’s Hospital of Jiangsu Province/Qidong Hospital Affiliated to Nantong University, No. 753 Jianghai Middle Road, Qidong, 226200 China; 3 Obstetrics and Gynecology Clinic, No. 963 Hospital, Jiamusi, 154003 China; 4 Obstetrics and Gynecology SAMII Medical Center in ShenZhen, No. 1, West Jinniu Road, Shenzhen, 518118 China; 5 Department of Gynecology, Maternal and Child Health Hospital of Liaocheng, No. 56 Changjiang Road, High-tech Zone, Liaocheng, 252000 China

**Keywords:** Cervical cancer, H1FX-AS1, miR-324-3p, DACT1, ceRNA

## Abstract

**Background:**

Cervical cancer (CC) is the 4th most common cancer-related death in gynecological cancer worldwide. It has been reported that many lncRNAs contribute to oncogenesis although the fundamental mechanisms are basically unknown. Here, we aimed to identify a novel lncRNA H1FX-AS1 and explore a ceRNA network in CC oncogenesis and progression.

**Methods:**

The expression level and the association with the prognosis of H1FX-AS1 in CC patients were analyzed based on Cancer Genome Atlas (TCGA) datasets, and further verified in 50 CC patients. The biological role of H1FX-AS1 was investigated in vitro and in vivo by over-expression of H1FX-AS1 in CC cells; the potential binding sites between H1FX-AS1 and miRNA, between miR-324-3p and DACT1 were predicted by LncBASE and Targetscan respectively, which were further verified by dual-luciferase reporter assay, RNA pull-down and point mutation; the relationship between genes was analyzed by Pearson correlation; the rescue experiments were used to further explore the involved molecular mechanism.

**Results:**

Lower H1FX-AS1 expression in CC tissues was found to be associated with the poor prognosis of CC patients. Over-expression of H1FX-AS1 inhibited CC cell proliferation, migration and invasion, while induced apoptosis by sponging miR-324-3p to up-regulate the DACT1 expression level.

**Conclusion:**

A novel lncRNA H1FX-AS1 was identified, which acted as a competing endogenous RNA (ceRNA) of miR-324-3p to inhibit DACT1 mediated CC progression. Therefore, H1FX-AS1 is a new prognostic predictor and targeting the factors in the H1FX-AS1/miR-324-3p/DACT1 axis is the novel potential therapeutic strategy for CC.

## Background

The cervical cancer (CC) ranks the 4th most frequently diagnosed cancer and the 4th leading cancer-related death in the females with a predictable 311,000 deaths and 570,000 cases in 2018 worldwide, although the screening and HPV vaccine have been applied; meanwhile, CC ranks the second in the mortality and incidence following the breast cancer in the lower Human Development Index settings, the most usually diagnosed malignancy in 28 countries and the leading cancer-related death in 42 countries [1], which rise severe public issues in woman health. Moreover, despite the advances in diagnosis and the aggressive treatment, the prognosis of CC is still quite unsatisfactory with a 5-year overall survival under 40% in nearly all countries, due to the cancer metastasis, recurrence, and unclear pathogenesis [2]. Therefore, it is critical to explore the novel molecular mechanism of CC oncogenesis, and to develop the new biomarkers for the diagnosis, prevention, treatment and prognosis of CC patients.

Non-coding RNAs (ncRNAs), including microRNA (miRNA) and long non-coding RNA (lncRNA), have been recently reported to be the important novel biomarkers in oncogenesis [3, 4]. LncRNAs include many transcript types with a minimum of 200 nucleotides (nt) without protein-coding ability [5–7]. The location relationship of lncRNAs with other genes includes introns, intergenic gene or overlapping in an antisense orientation, while the lncRNA expression is usually tissue specific, involving in diverse genetic processes, for example, the mRNA stability, transcription and posttranslational modification [8–10], thus contributes to the oncogenesis and tumor progression [11]. Competing endogenous RNA (ceRNA) has been noted to be a new regulatory mechanism model and recognized to mediate the lncRNA function. In which, the expression levels of both mRNA and lncRNA are regulated by competitively binding to the shared miRNAs and degradation of the target mRNAs [12].

By searching the literatures we found the function of H1FX-AS1 in CC was not reported. According to the TCGA datasets we identified that H1FX-AS1 was down-regulated in the CC tissues, which was confirmed to be associated with the overall survival in CC patients after our further analysis and confirmed the identical expression level in our pretest experiments. By the bioinformatics prediction, miR-324-3p was designated as the potential target of H1FX-AS1, which could potentially bind to DACT1, therefore, miR-324-3p was investigated as a prospective ceRNA of H1FX-AS1, and the potential of H1FX-AS1/miR-324-3p/DACT1 ceRNA network in CC progression as the novel prognostic predictors, the prevention and therapeutic targets for the CC patients was investigated in our current work.

## Patients and methods

### Patient specimens

We collected the CC and self-matched nearby normal cervical tissues from 50 CC patients during the surgery treatment, and stored at − 80 °C after liquid nitrogen freezing.

### Informed consents

The written informed consents were provided by all the CC patients before the tissues were collected. All experiments were agreed by the Ethics Committees of all participation hospitals following the Helsinki Declaration.

### Cell culture

Four CC (SiHa, C-4I, HeLa and C-33A) and a normal cervical epithelial (End1/E6E7) cell lines were provided by the Committee on Type Culture Collection of the Chinese Academy of Sciences (Shanghai, China), which were maintained in the Macoy’s 5A medium containing the streptomycin (100 mg/mL), penicillin (100 IU/mL) and FBS (10%), and a humidified 5% CO_2_ incubator at 37 °C.

### Reagents

Propidium iodide, APC-Annexin V and protease inhibitor cocktail were provided by Sigma (St. Louis, MO, USA); CCK-8 test kit was provided by Dojindo Corp (Kyushu, Japan); lipofectamine 3000 and Trizol were from Thermo Fisher Scientific (Waltham, MA, USA); Apoptosis Detection Kit was from Beyotime (Nanjing, China); Dual-Luciferase Reporter Assay System was provided by Promega (Madison, WI, USA); matrigel was from BD (New Jersey, USA); Macoy’s 5A medium was from Gibco (Rockford, MD, USA); SurePrep™ Nuclear or Cytoplasmic RNA Purification Kit was from Fisher BioReagents^®^ (Fair Lawn, NJ, USA). Antibodies against the Bcl-2 (# 3498), GAPDH (#2118) and Cleaved caspase3 (# 9661S) were obtained from Cell Signaling Technology, Inc. (Danvers, MA, USA); antibody against the DACT1 was from Novus Biologicals, LLC (Centennial, CO, USA).

### Bioinformatics analysis for target gene identification

LncBASE was applied to predict the miRNAs potentially being sponged by H1FX-AS1. Targetscan was applied to predict the prospective binding sites of miR-324-3p with DACT1.

### Lentiviral vector, siRNA, inhibitors, mimics and luciferase activity assay

The over-expression and control lentivirus vectors were bought from Hanbio (Shanghai, China).The genetically modified stable cells were established after the routine infection and selection following the manufacturer’s instructions. SiRNA, mimics and the negative control (NC) were provided by GenePharma (Shanghai, China). Lipofectamine 3000 was used for target gene transfection following the manufacturer’s instructions.

#### Dual-luciferase reporter assay

According to the bioinformatics prediction, miR-324-3p was designated as the potential target of H1FX-AS1, which could potentially bind to DACT1. The pmirGLO Dual-Luciferase miR Target Expression Vector was then applied to verify the direct interaction between the candidate miR-324-3p and the H1FX-AS1 (or DACT1). The wild-type (WT) reporter construct pmirGLO/H1FX-AS1 (or DACT1) and the mutant reporter construct pmirGLO/H1FX-AS1-mut (or DACT1-mut) was co-transfected with miR-324-3p mimic or miR-NC (negative control) in SiHa and HeLa cells. The Firefly luciferase levels were checked by the microplate reader (a luminometer) 48 h after the transfection, and normalized to the Renilla luciferase activity, which was used as the final luciferase activity of the tested report gene.

### Cytoplasmic and nuclear RNA fraction isolation

A SurePrep™ Nuclear or Cytoplasmic RNA Purification Kit (Cat. # BP2805-25) was used to isolate the nuclear and cytoplasmic RNA fractions, following the manufacture’s protocol. Briefly, the monolayer SiHa and HeLa cells were collected respectively and lysed using the Lysis Solution. The lysate was centrifuged for 3 min at 12,000 RPM. The supernatant containing the cytoplasmic RNA was transferred into an RNase-free tube, and retained the pellet containing the nuclear RNA, followed by binding the cytoplasmic and nuclear RNAs to the columns respectively. Then the columns were washed and RNAs were eluted. The cellular sublocalization expression of H1FX-AS1 was tested with RT-qPCR. U6 and GAPDH were used as the internal control.

### Total RNA isolation and RT-qPCR analysis

The purity and concentration of the total RNA isolated with Trizol were evaluated with ND-1000 model NanoDrop (Thermo Fisher Scientific, MA, USA). PrimeScrip™ RT Master Mix for reverse transcription, SYBR Green Premix Ex Taq™ II for cDNA amplification and thermo recycler AB7300 (Applied Biosystems, CA, USA) with the TaqMan Universal PCR Master Mix for RT-qPCR assay were performed respectively. The relative gene expression was represented by 2^−ΔΔ*C*t^ method.

### Apoptosis evaluation

The apoptosis was determined using the Apoptosis Detection Kit following the flow cytometric assay with the Cell Quest software (version 1.2.2, Becton Dickinson & Company, Franklin Lakes, NJ, USA).

### Evaluation of colony formation ability

The 7-day-cultured target cells, in a 6-well plate with the original concentration of 1000 cells/well, were fixed for 10 min in 4% paraformaldehyde, followed by staining for 5 min with 0.5% crystal violet. A light microscope (Olympus, Japan) and ImageJ were then applied for colony image and number collection.

### Cell viability evaluation

The CCK-8 kit was used to evaluate the cell viability according to the manufacturer’s instructions. Briefly, a 10 μL of CCK-8 reagent was added into the culture medium of cells to be tested in a 96-well plate, and cultured at 37 °C in dark for 2 h. The optional density (OD) was estimated at 450 nm using a 680 Microplate Reader (Bio-Rad, Hercules, CA).

### Wound healing assay for cell migration ability evaluation

A 10-µl pipette tip was used to carefully scratch a wound at the middle of the wells of the 6-well plate with 100% confluent cells. The cells were washed with sterile PBS and then cultured in fresh serum-free medium at 37 °C for 24 h to get the cell migration images at 0 and 24 h under an inverted light microscope (Olympus, Japan).

### Transwell invasiveness ability assay

A 24-well transwell with 8.0 μm pore size (Corning Costar, USA) and coated Matrigel was used to estimate the invasiveness ability. In brief, 500 μL of medium with 10% FBS was loaded into the lower chamber; 3 × 10^5^ cells in 200 μl of serum-free medium was loaded into the upper chamber for 48 h culture; the remained cells on the opposite side of the upper chamber after removing those on the upper side using a cotton swab, which represented the invaded cells, were fixed in 4% paraformaldehyde followed by 0.1% crystal violet staining.

### Total protein isolation and western blot assay

Cells to be tested were lysed with the NP40 lysis buffer (10% glycerol, 5 mM EDTA, 0.1% NP-40, 150 mM NaCl and 50 mM Tris–HCl) containing the protease inhibitor cocktail. Same amounts of proteins were separated on a 8% SDS-PAGE gel, transferred onto the PVDF membranes and immunoblotted with primary antibodies at 4 °C overnight, followed by secondary antibody culture at room temperature for 1 h.

Antibodies against the Bcl-2(1:1000),GAPDH (1:1000) and Cleaved caspase 3 (1:1000); and antibody against the DACT1 (1:1000) were used.

### Animal experiments

Forty female 6-week-old BALB/c nude mice were provided by Beijing HFK Bioscience Co. Ltd. (Beijing, China), divided into four groups (n = 10) and housed in the animal room of Nantong University. All animal experimental processes were approved by the Animal Experimental Ethics Committee of Qidong Hospital Affiliated to Nantong University. The SiHa and HeLa cells over-expressed H1FX-AS1 or the NC (1 × 10^7^ cells/mouse/200 μl of PBS) were injected subcutaneously on the back of the mice. The tumor volume was measured every week with a vernier caliper. Nude mice were sacrificed 35 days after inoculation, and the tumor weight was measured.

### Statistics process

The data were presented as mean ± SD. Two or multiple group comparison was performed by Student’s t-test or one-way ANOVA respectively with the SPSS 20.0 (IBM, Chicago, USA). Survival curves were achieved by Kaplan–Meier analysis and compared with log-rank test. The relationship between genes was evaluated by Pearson Chi square analysis. A *p *< 0.05 was statistically significant.

## Results

### Down-regulated H1FX-AS1 and up-regulated miR-324-3p were identified in both CC patients and cells, and low H1FX-AS1 expression was correlated with poor prognosis of CC patients

**Fig. 1:**
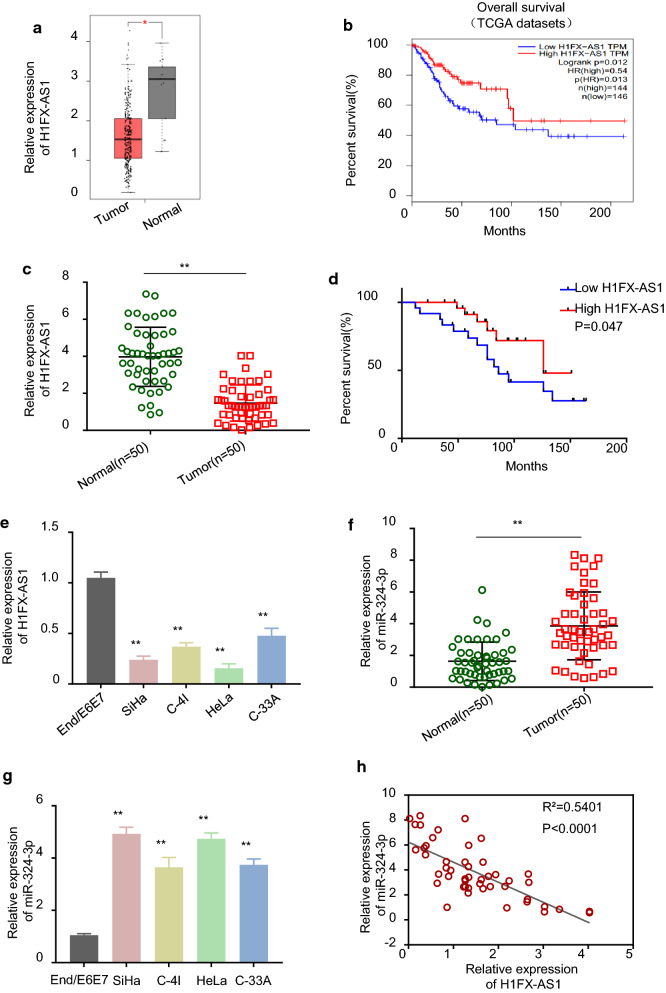
H1FX-AS1 expression was significantly down-regulated and miR-324-3p was significantly up-regulated in CC tissues and cell lines. **a** H1FX-AS1 expression was statistically significantly lower in the CC (T) tissues than in the normal (N) tissues obtained by analyzing the TCGA datasets; **b** CC patients with low H1FX-AS1 expression had a poor prognosis from the TCGA datasets analyzed by GEPIA; **c** H1FX-AS1 expression was statistically significantly lower in the CC tissues than in the paired normal adjacent tissues from 50 CC patients analyzed by RT-qPCR; **d** the survival curve of the 50 CC patients with H1FX-AS1 high or low expression by KM-plotter plots, indicating poor prognosis in CC patients with H1FX-AS1 low expression; **e** H1FX-AS1 expression was statistically significantly lower in the CC cells (SiHa, C-4I, HeLa and C-33A) than in the normal human cervical epithelial End1/E6E7 cells analyzed by RT-qPCR, and the difference was most significant in the SiHa and the HeLa cells; **f** miR-324-3p expression was statistically significantly higher in the CC tissues than in the paired normal adjoining tissues from 50 CC patients analyzed by RT-qPCR; **g** miR-324-3p expression was statistically significantly higher in the CC cells (SiHa, C-4I, HeLa and C-33A) than in the normal human cervical epithelial End1/E6E7 cells analyzed by RT-qPCR; **h** Pearson correlation analysis of the association between miR-324-3p with H1FX-AS1 in the CC tissues from the 50 CC patients (same samples as in **c**, and **f**). **p < 0.01

To explore the expression profile and the clinical function of H1FX-AS1 in CC patients, we analyzed the Cancer Genome Atlas (TCGA) datasets, and the results demonstrated that H1FX-AS1 expression was statistically significantly lower in the CC (T) tissues than in the normal (N) tissues from the TCGA datasets (Fig. 1a, *p *< 0.05), and analysis by GEPIA confirmed that the CC patients with low H1FX-AS1 expression level had a poor prognosis (Fig. 1b, *p *< 0.05). To further verify the above results, we collected the CC and the self-matched adjacent normal cervical tissues, as well as the clinicopathological characteristics (Table 1) from 50 CC patients, as we can see in Fig. 1c, H1FX-AS1 expression level was statistically significantly lower in the CC tissues than in the paired normal adjacent tissues analyzed by RT-qPCR (*p *< 0.01); after the 50 CC patients were divided into the H1FX-AS1 low expression level group (n = 25) and the H1FX-AS1 high expression level group (n = 25) with the cut-off value of H1FX-AS1 median expression level in CC tissues, the survival curves of the CC patients with H1FX-AS1 high expression level and H1FX-AS1 low expression level were plotted by Kaplan–Meier analysis, which showed a poor prognosis in CC patients with H1FX-AS1 low expression level; the relationship between H1FX-AS1 expression level and the clinicopathological characteristics of CC patients analyzed by Chi square test showed that the H1FX-AS1 expression was statistically significantly related to the tumor size, the TNM stage, and the lymph node metastasis (p < 0.05), while not related to the patient age and the tumor differentiation (Table 1 and Fig. 1d). The down-regulated H1FX-AS1 expression level was also verified in CC cells, as shown in Fig. 1e, the expression level of H1FX-AS1 was statistically significantly lower in all tested CC cells (SiHa, C-4I, HeLa and C-33A) versus the End1/E6E7 cells (*p *< 0.01). Meanwhile, the miR-324-3p expression level was detected by RT-qPCR in both the CC tissues and cells, our results showed that miR-324-3p expression level was statistically significantly higher in the CC tissues than in the paired normal adjacent tissues from 50 CC patients (Fig. 1f, *p *< 0.01); miR-324-3p expression level was statistically significantly higher in the CC cells (SiHa, C-4I, HeLa and C-33A) than in the normal human cervical epithelial End1/E6E7 cells (Fig. 1g, *p *< 0.01). In additional, Pearson correlation analysis demonstrated that miR-324-3p showed a negative correlation with H1FX-AS1 expression level in the CC tissues from the 50 CC patients (Fig. 1h, *p *< 0.001). These results showed that H1FX-AS1 was down-regulated and miR-324-3p was up-regulated in both the CC patients and cells, down-regulated H1FX-AS1 expression level was correlated with the poor prognosis of CC patients.

**Table 1:** The relationship between H1FX-AS1 expression and the clinicopathological characteristics of CC patients was analyzed by Chi square test

Clinicopathological characteristics	Total	High expression	Low expression	X^2^	*P* value
Age					
≤ 35	28	12	16	1.299	0.254
> 35	22	13	9		
Differentiation					
High	17	8	9	0.111	0.946
Moderate	14	7	7		
Poor	19	10	9		
Tumor size (cm)					
< 4	28	18	10	5.195	0.023
≥ 4	22	7	15		
TMN stages					
I	15	12	3	14.274	0.003
II	11	7	4		
III	13	5	8		
IV	11	1	10		
Lymph node metastasis					
Positive	21	6	15	6.650	0.010
Negative	29	19	10		

The 50 CC patients were divided into H1FX-AS1 low expression group (n = 25) and H1FX-AS1 high expression group (n = 25) with the cut-off value of H1FX-AS1 median expression in CC tissues

### Over-expression of H1FX-AS1 inhibited proliferation, migration, and invasion, while induced apoptosis in CC cells

**Fig. 2:**
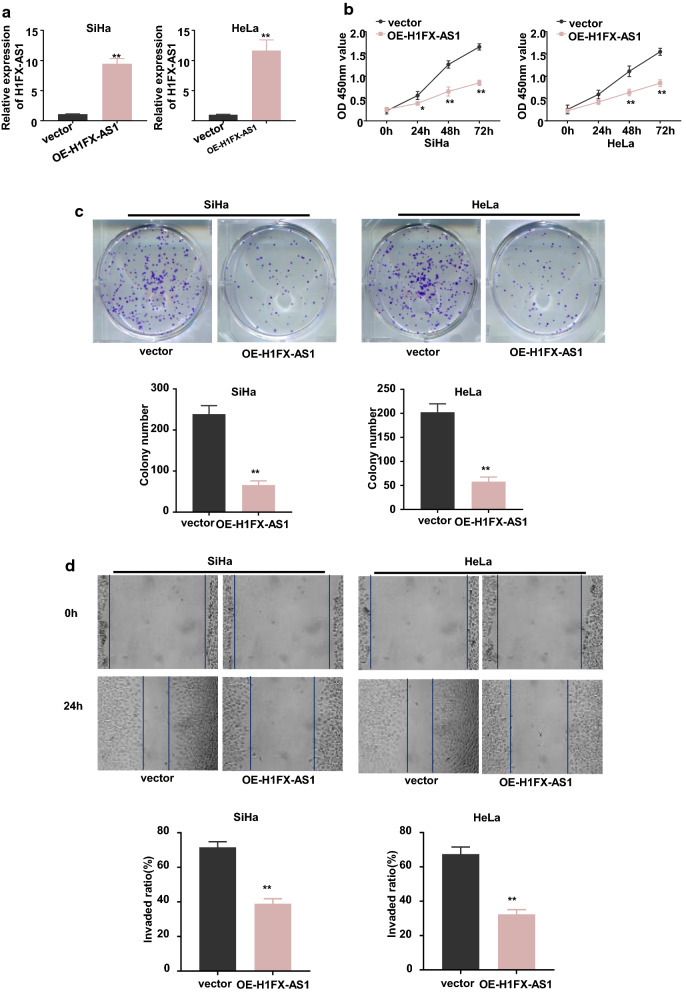

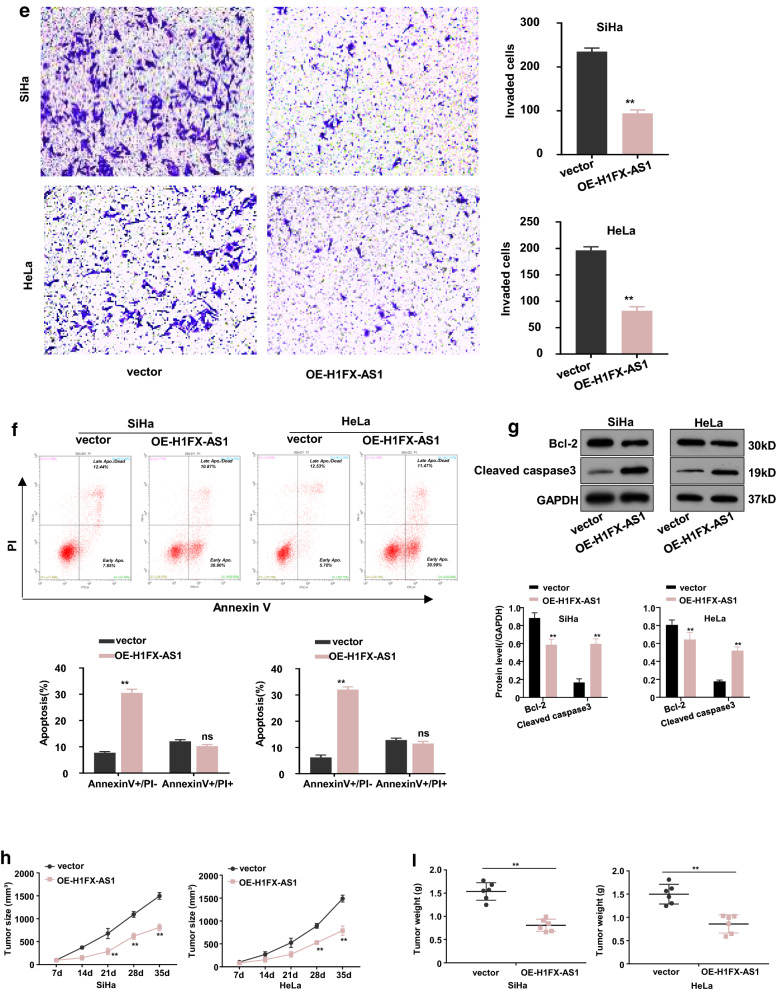
Over-expression of H1FX-AS1 inhibited proliferation, migration and invasion,while induced apoptosis in CC cells both in vivo and in vitro. To investigate the influence of H1FX-AS1 expression in CC development, H1FX-AS1 was over-expressed in SiHa and HeLa cells (the two tested cell lines showing the lowest H1FX-AS1 expression), when RT-qPCR analysis confirmed that H1FX-AS1 was successfully over-expressed in both the SiHa and HeLa cells **a**, the following phenotypes were further estimated in the SiHa and HeLa cells over-expressed H1FX-AS1 (OE-H1FX-AS1): **b** cell viability, **c** clone formation ability (images: upper panel; quantification: lower panel), **d** cell migration(images: upper panel; quantification: lower panel), **e** cell invasion(images: left panel; quantification: right panel), **f** apoptosis (images: upper panel; quantification: lower panel), **g** apoptosis-related proteins (images: upper panel; quantification: lower panel). OE-H1FX-AS1 in both the SiHa and HeLa cells inhibited the xenograft tumor growth: **h** growth curve (tumor volume) analysis of the xenograft tumors with H1FX-AS1 over-expressed or the control vector transfected SiHa or HeLa cells; **i** the average tumor weights between the over-expressed or the control vector transfected SiHa or HeLa cell groups. **p < 0.01

The successful over-expression of H1FX-AS1, in the SiHa and HeLa cells with the lowest H1FX-AS1 expression, was determined by RT-qPCR analysis (Fig. 2a, p < 0.01). We identified that over-expression of H1FX-AS1 significantly reduced the viability tested by the CCK-8 assay (Fig. 2b), the clone formation ability (Fig. 2c), cell migration tested by the wound healing assay (Fig. 2d) and invasive potential tested by the transwell analysis (Fig. 2e), while induced apoptosis tested by the flow cytometric analysis (Fig. 2f). In addition, the expression level of cleaved caspase 3 (the active apoptotic effector protein) was increased; while the anti-apoptotic protein Bcl-2 was decreased by over-expression of H1FX-AS1 in these two cell lines detected by western blot assay (Fig. 2g). A xenograft tumor model was then made to further confirm the effect on tumor growth after subcutaneous inoculation with H1FX-AS1 stably over-expressed SiHa or HeLa cells. We demonstrated that the tumor proliferative activity, including the tumor volume and tumor weight, was decreased in H1FX-AS1 over-expressed group versus the control vector group (Fig. 2h, *p *< 0.01). Collectively, our results demonstrated that H1FX-AS1 acted as a tumor-suppressor to inhibit the tumorigenesis of CC.

### H1FX-AS1 served as a competing endogenous RNA to sponge miR-324-3p in CC cells

**Fig. 3:**
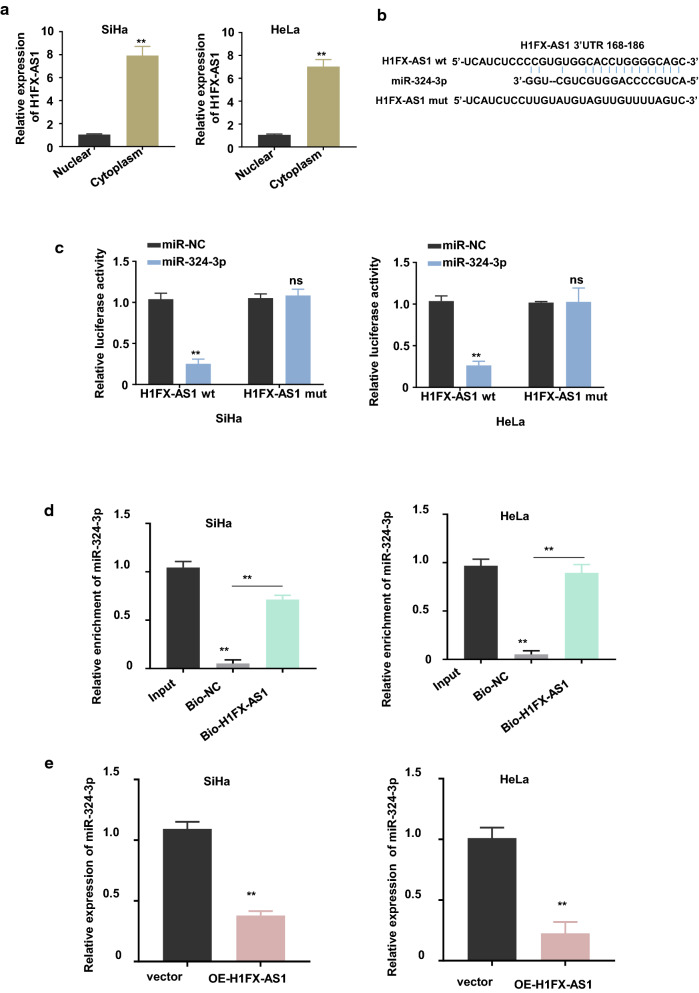
H1FX-AS1 acted as a sponge for miR-324-3p. **a** Cellular sublocalization of the H1FX-AS1 in the SiHa and HeLa cells were determined by the nuclear and cytoplasmic separation experiments, U6 and GAPDH are used as internal references for nuclear and cytoplasmic expression, respectively. The potential binding sites of H1FX-AS1 with miR-324-3p were identified: the schematic representation of the potential binding sites of H1FX-AS1 with miR-324-3p predicted by lncBASE (**b**), which was verified by double luciferase reporter assay in the SiHa (left panel) and HeLa (right panel) cells after co-transfection of H1FX-AS1 WT or mutated reporter with or without miR-324-3p mimics (**c**); miR-324-3p in the cell lysates of SiHa (left panel) and HeLa (right panel) cells was further pulled down and enriched with biotin labeled H1FX-AS1 specific probe (**d**); the miR-324-3p expression level was confirmed to be negatively related with the H1FX-AS1 expression level after checking the miR-324-3p expression level in the SiHa (left panel) and HeLa (right panel) cells with (OE-H1FX-AS1) or without (vector) H1FX-AS1 over-expression (**e**). **p < 0.01

Emerging evidences have reported that cytoplasmic lncRNAs predominantly serve as the competing endogenous RNAs (ceRNAs) through sponging the specific miRNAs that degrade the target genes [13, 14]. Given that H1FX-AS1 is a novel identified lncRNA, we performed a nuclear and cytoplasmic separation followed by RT-qPCR assay to determine the cellular sublocalization expression level of H1FX-AS1 in SiHa and HeLa cells. The results showed that the cytoplasmic H1FX-AS1 was predominant versus the nuclear fraction (Fig. 3a, *p *< 0.01), therefore, we hypothesized that H1FX-AS1 may be a sponge of miRNAs to rescue the expression of genes targeted by miRNAs in CC development. Herein, we investigated the potential miRNAs that could bind to H1FX-AS1 in CC cells. After the bioinformatics prediction by searching the lncBASE (http://carolina.imis.athena-innovation.gr/diana_tools/web/index.php?r=lncbasev2%2Findex-predicted), miR-324-3p was found to be potentially bound to H1FX-AS1, and the schematic representation of the predicted binding sites of H1FX-AS1 with miR-324-3p was shown in Fig. 3b. To verify the true interaction between H1FX-AS1 and miR-324-3p, we subcloned the H1FX-AS1, with WT or mutated reporter gene, into the pmirGLO dual luciferase reporter vector. Dual luciferase assay showed that over-expression of miR-324-3p with the mimics inhibited the luciferase activities of the wild-type H1FX-AS1 reporter gene in both the SiHa and HeLa cells (p < 0.01), while this effect was neutralized after the predicted binding sites between H1FX-AS1 and miR-324-3p were mutated (Fig. 3c). The direct binding between H1FX-AS1 and miR-324-3p was further confirmed using RNA in vivo precipitation (RIP), which showed that, versus the control oligo probe, more miR-324-3p was statistically significantly pulled down by the H1FX-AS1 probe in both the SiHa and HeLa cells (Fig. 3d, *p *< 0.01). Meanwhile, we found that H1FX-AS1 over-expression down-regulated miR-324-3p expression by over 50% in both the SiHa and HeLa cells (*p* < 0.01, Fig. 3e). These results showed that the cytoplasmic *H1FX*-*AS1 served as a ceRNA to sponge the miR*-*324*-*3p in CC cells.*

### H1FX-AS1 sponged miR-324-3p to up-regulate dishevelled binding antagonist of beta catenin 1(DACT1) in CC cells

**Fig. 4:**
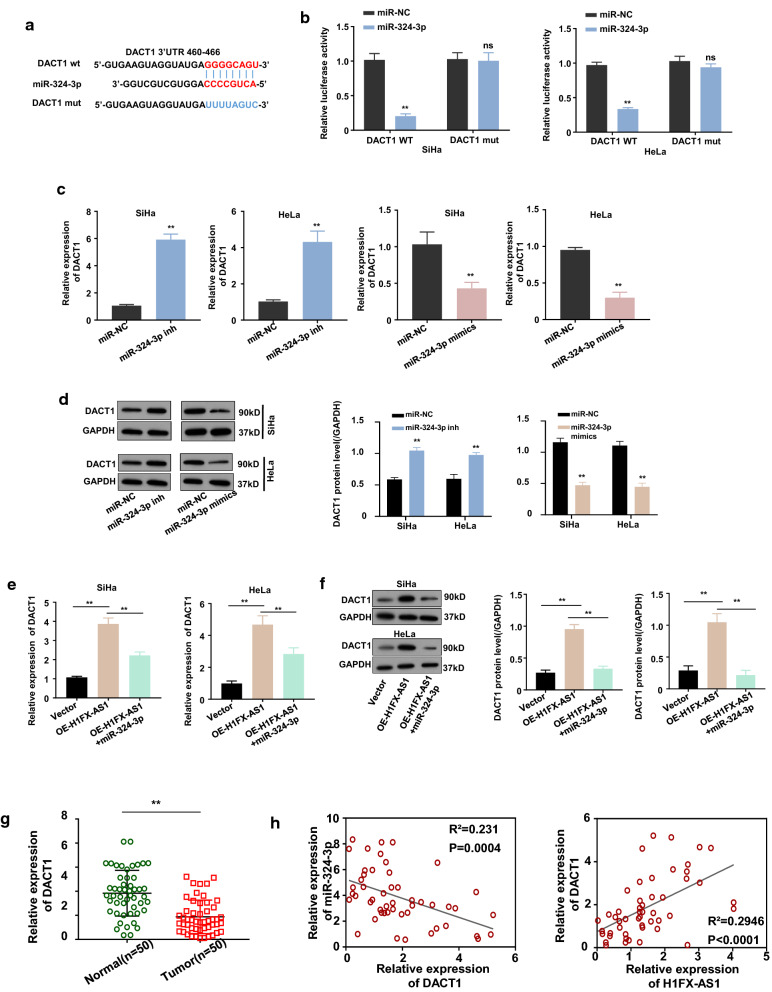
H1FX-AS1 promoted DACT1 expression via sponging miR-324-3p. The schematic representation of the potential binding sites of miR-324-3p in the 3′ UTR of DACT1 predicted by targetscan (**a**), which was further confirmed by the dual-luciferase reporter assay of the SiHa (**b**, left panel) and HeLa (**b**, right panel) transfected by the WT or mutated DACT1-3′ UTR reporter with or without co-transfection of miR-324-3p; the mRNA (**c**) and the protein (**d**, images: left panel; quantification: right panel) expression levels of DACT1 in the SiHa and HeLa cells after miR-324-3p was silenced (miR-324-3p mimics) or over-expressed (miR-324-3p mimics); the mRNA (**e**) and the protein (**f**, images: left panel; quantification: right panel) expression levels of DACT1 in the SiHa and HeLa cells after H1FX-AS1 over-expression with or without miR-324-3p co-transfection; **g** the relative expression levels of DACT1 in paired CC and nearby normal tissues from 50 patients (same samples as in Fig. 1c, f) detected by RT-qPCR; **h** Pearson correlation analysis of the associations between DACT1 with H1FX-AS1 (left panel) and miR-324-3p (right panel) in the CC tissues from 50 CC patients (same samples as in Fig. 1c, f). **p < 0.01

DACT1 is a Wnt-pathway inhibitor, it has been reported that miR-324-3p activated Wnt/β-catenin signaling via targeting DACT1 to promote hepatocellular carcinoma growth [15], meanwhile, we predicted the binding sites of miR-324-3p in the 3′ non-coding region(UTR) of DACT1 by targetscan (Fig. 4). Therefore, we hypothesized that miR-324-3p may directly bind to and inhibit the DACT1 expression. We then subcloned the DACT1 reporter gene with WT or mutated 3′UTR of DACT1 into the pmirGLO dual luciferase reporter vector. Dual luciferase assay displayed that over-expression of miR-324-3p with the mimics inhibited the luciferase activity of the wild-type DACT1 reporter gene in both the SiHa and HeLa cells (p < 0.01), while this effect was diminished after the predicted binding sites between miR-324-3p and the 3′UTR of DACT1 were mutated (Fig. 4b), indicating the existence of the direct interaction between the miR-324-3p and the 3′UTR of DACT1. This direct interaction was further confirmed by measure the mRNA and protein expression levels of DACT1 in the SiHa and HeLa cells after over-expressing or silencing miR-324-3p, which showed that knockdown (miR-324-3p inh) or over-expression (miR-324-3p mimics) of miR-324-3p respectively increased or decreased the mRNA (Fig. 4c, *p* < 0.01) and protein (Fig. 4d, *p* < 0.01) expression levels of DACT1. Since H1FX-AS1 competes the binding to the 3′UTR of DACT1 with miR-324-3p, we wondered whether H1FX-AS1 modulates miR-324-3p-mediated inhibition of DACT1. To find out whether H1FX-AS1 sponges miR-324-3p, thus to regulate DACT1 expression in SiHa and HeLa cells, we first detected the mRNA and the protein expression levels of DACT1 in the SiHa and HeLa cells after H1FX-AS1 over-expression with or without miR-324-3p co-transfection by RT-qPCR and Western blot assays, our results revealed that H1FX-AS1 over-expression significantly up-regulated DACT1 expression at both the mRNA (Fig. 4e, *p* < 0.01) and protein (Fig. 4f, *p* < 0.01) levels in the SiHa and HeLa cells, and these effects were sufficiently reversed by co-transfection of the miR-324-3p mimics. DACT1 expression level was further quantified with RT-qPCR in the CC tissues from 50 CC patients, which proved that DACT1 expression was significantly decreased in the CC tissues versus the nearby normal tissues (Fig. 4g, *p *< 0.01). Meanwhile, DACT1 expression level in the CC tissues from 50 CC patients was found to be statistically significantly and positively related to the H1FX-AS1 expression level (Fig. 4h, the left panel, *p *< 0.001), while statistically significantly and negatively related to the miR-324-3p expression level by the Pearson correlation analysis (Fig. 4h, the right panel, *p *< 0.001). These results showed that DACT1 was up-regulated when the miR-324-3p was sponged by the H1FX-AS1 in the CC cells.

### The miR-324-3p over-expression or DACT1 knockdown blocked H1FX-AS1 induced inhibition of the aggressive behaviors in CC cells

**Fig. 5:**
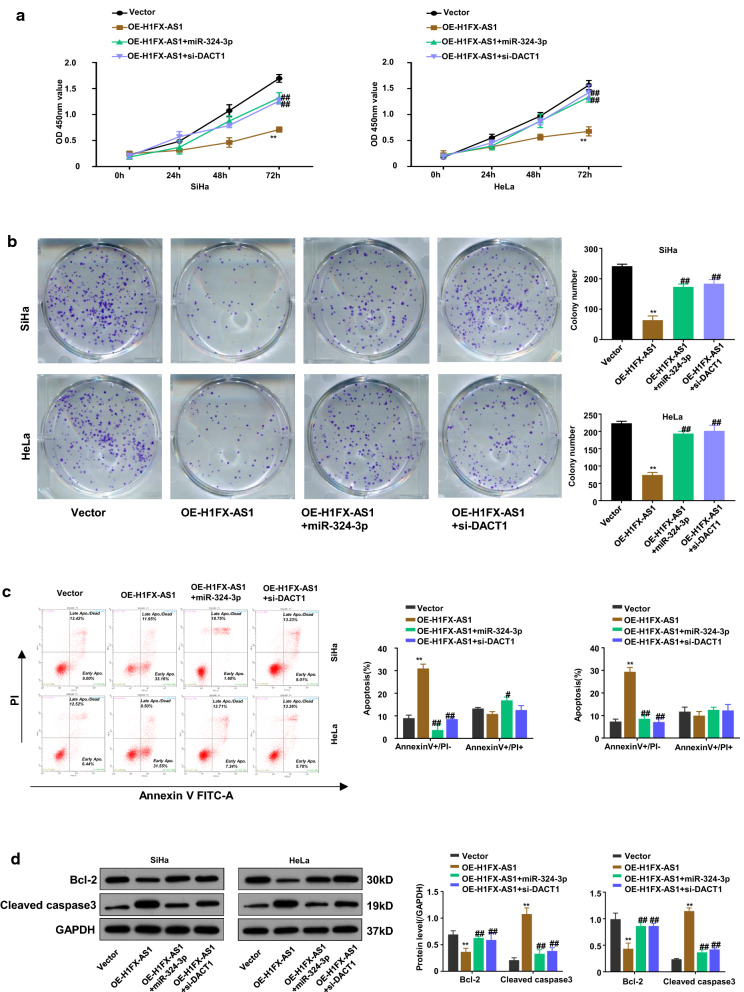

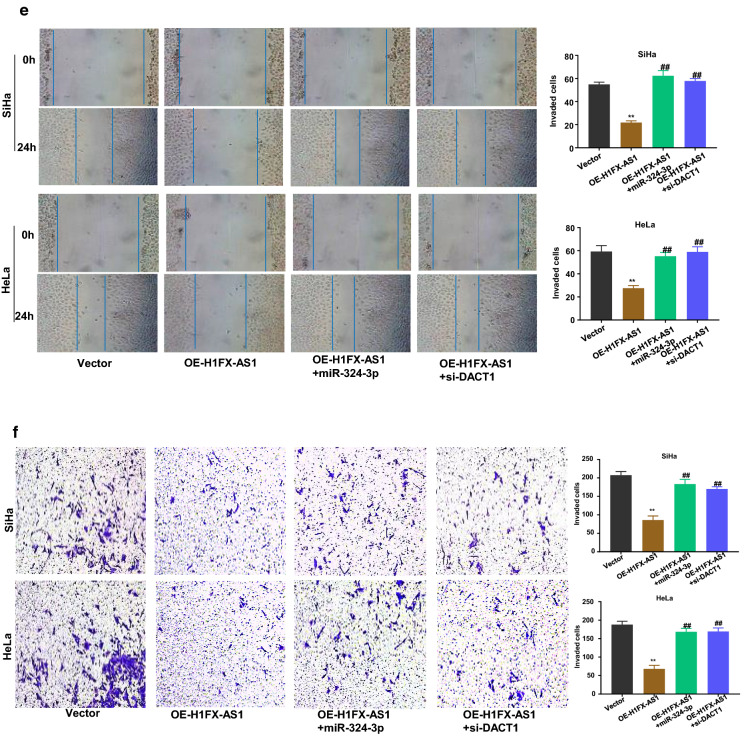
MiR-324-3p over-expression or DACT1 knockdown blocked H1FX-AS1-induced inhibition of the aggressive behaviors in CC cells. To further determine the functional effect of the H1FX-AS1/miR-324-3p/DACT1 axis in the CC cells, the **a** cell viability, **b** clone formation ability, **c** apoptosis, **d** apoptosis-related proteins, **e** cell migration, **f** cell invasion in SiHa and HeLa cells after H1FX-AS1 over-expression (OE-H1FX-AS1) with or without miR-324-3p over-expression by miR-324-3p mimics (miR-324-3p) or DACT1 knockdown (vector, OE-H1FX-AS1, OE-H1FX-AS1 + miR-324-3p, OE-H1FX-AS1 + si-DACT1) by small interfering RNA (si-DACT1) were investigated, respectively. **p < 0.01, ^##^*p* < 0.01

To further confirm the functional effect of the H1FX-AS1/miR-324-3p/DACT1 axis in SiHa and HeLa cells, the cell viability, clone formation ability, apoptosis, apoptosis-related proteins, migration and invasion in SiHa and HeLa cells after H1FX-AS1 over-expression (OE-H1FX-AS1) with or without miR-324-3p over-expression by miR-324-3p mimics (miR-324-3p) or DACT1 knockdown by small interfering RNA (si-DACT1) were further investigated, respectively. The results showed that over-expression of H1FX-AS1 statistically significantly reduced the OD values of the SiHa and HeLa cells at 450 nm wavelength, detected by CCK-8 assay at different time points (0 h, 24 h, 48 h and 72 h), and this effect was partially rescued when the miR-324-3p mimics or si-DACT1 was co-transfected (Fig. 5a, p < 0.01); the clone formation abilities of SiHa and HeLa cells were also statistically significantly reduced after H1FX-AS1 over-expression, which was partially rescued when the miR-324-3p mimics or si-DACT1 was co-transfected (Fig. 5b, p < 0.01); the apoptotic rates of SiHa and HeLa cells, detected by the flow cytometric analysis, were statistically significantly increased by over-expression of H1FX-AS1, while this effect was partially reduced when co-transfected with miR-324-3p mimics or si-DACT1(Fig. 5c, p < 0.01); the western blot assay showed that the increased expression of cleaved Caspase 3 and the decreased Bcl-2 expression after over-expression of H1FX-AS1 were partially rescued when the miR-324-3p mimics or si-DACT1 was co-transfected in SiHa and HeLa cells (Fig. 5d); the wound healing assay showed that the reduced cell migration abilities of SiHa and HeLa cells after over-expression of H1FX–AS1 were partially recovered when co-transfected with miR-324-3p mimics or si-DACT1 (Fig. 5e); the transwell assay showed that the reduced cell invasive abilities of SiHa and HeLa cells after over-expression of H1FX-AS1 were also statistically significantly reversed when co-transfected with the miR-324-3p mimics or si-DACT1 (Fig. 5f). Collectively, all of these observations indicate that H1FX-AS1 sponges miR-324-3p to activate DACT1, thus to inhibit the highly aggressiveness of the CC cells. These results showed that over-expressed miR-324-3p or inhibited DACT1 blocked the preventative effect of H1FX-AS1 in CC development.

## Discussion

More than 80% of genes in the human genome are non-coding ones including the lncRNAs, which were originally believed to be the transcriptional “noise” and have been recently reported functional necessary molecules [8, 16].

In current study, we first identified H1FX-AS1, a novel lncRNA, was down-regulated in the CC cells and tissues, also the TCGA datasets, suggesting a prospective tumor suppressor gene in CC.

Kaplan–Meier analysis further confirmed that the low H1FX-AS1 expression indicated a poor prognosis in CC patients, which was significantly related to the tumor size, TNM stage, and lymph node metastasis, suggesting a potential prognostic marker or therapeutic target for CC patients.

The slight difference in the clinical survival rate between the TCGA dataset with our local patients may be caused by the varied regions where the patients came from. Patients in the TCGA database were global, while our local patients only included those from China. However, the results from these two sets showed the same pattern, thus we presented both in our current work to solidate the evidences of our conclusion.

Different molecular mechanisms can be applied to regulate the gene and protein functions since the cellular lncRNAs distribute in cytoplasm and/or nucleus, therefore, abnormal lncRNAs may trigger various cellular dysfunction, such as malignant transformation [17]. Our results found that H1FX-AS1 was a cytoplasmic lncRNA. Cumulative studies have reported that cytoplasm lncRNAs predominantly serve as ceRNAs by sponging the specific miRNAs and protect miRNAs from binding to and degrading the target genes [12–14]. Therefore, the competing miRNA being sponged by H1FX-AS1 and the target mRNA regulated by the miRNA were further explored. Bioinformatics analysis showed that miR-324-3p was the potential miRNA sponged by H1FX-AS1, which was further confirmed by Pearson correlation, dual luciferase reporter gene and RIP assays. Bioinformatics analysis and literature both revealed that DACT1 was the target gene of miR-324-3p, which was further verified by the Pearson correlation assay, and the function rescue experiments. Thus, in this study, we also revealed a novel mechanism that H1FX-AS1 served as a ceRNA of miR-324-3p to up-regulate the DACT1 expression. Considering the important function of Wnt-signaling pathway in CC development and DACT1 is the inhibitor of the Wnt-signaling, our results for the first time found out the therapeutic significance of H1FX-AS1/miR-324-3p/DACT1 axis in CC patients.

## Conclusion

Our current study revealed that H1FX-AS1 expression was significantly down-regulated in CC patients and cells. Lower H1FX-AS1 expression in CC tissues was found to be associated with the poor prognosis of CC patients. Functionally, H1FX-AS1 inhibited CC cell growth, clone formation, migration and invasion, while induced apoptosis by sponging miR-324-3p to up-regulate the DACT1 expression. Therefore, our findings highlighted the prospective role of H1FX-AS1 as a new prospective tumor suppressor gene and a prognostic predictor in CC patients, and a ceRNA of miR-324-3p to inhibit DACT1 mediated CC progression. As a result, targeting H1FX-AS1/miR-324-3p/DACT1 axis would be a novel therapeutic strategy for CC.

## Data Availability

All data supporting the results of this work are available from the corresponding author upon request. These data are not available in public because of the ethical restrictions or privacy.
